# Non-Targeted Metabolomics Analysis of Small Molecular Metabolites in Refrigerated Goose Breast Meat

**DOI:** 10.3390/vetsci11120637

**Published:** 2024-12-09

**Authors:** Dongzhi Miao, Xuebei Wu, Kui Zuo, Jing Chen, Ying Wang, Junhua Pu, Haiming Yang, Zhiyue Wang

**Affiliations:** 1 College of Animal Science and Technology, Yangzhou University, Yangzhou 225009, China; miaodongzhi@163.com (D.M.); 17849897240@163.com (X.W.); 13376061975@163.com (K.Z.); cj82955@163.com (J.C.); dkwzy@263.net (Z.W.); 2 Jiangsu Institute of Poultry Sciences, Poultry Institute, Chinese Academy of Agricultural Sciences, Yangzhou 225009, China; apu123@sina.com

**Keywords:** Yangzhou goose, frozen storage, non-targeted metabolomics, random forests, meat quality

## Abstract

Poultry represents a rich source of multiple nutrients. Refrigeration is commonly employed for poultry preservation, although extended storage duration can adversely affect the meat quality. Current research on this topic has focused on the analysis of biochemical indices in chilled goose meat, with limited information on changes in metabolites that influence the quality of the meat during storage. This study used non-targeted metabolomics and the random forest algorithm to investigate metabolite changes in goose meat over an extended storage period. The results showed a significant change in the composition of the meat as the duration of storage increased, with the identification of 121 distinct metabolites. Further analysis identified 18 metabolites that could be used as indicators of the degradation of carbohydrates, amino acids, nucleotides, and lipids. These metabolites could be used as markers to monitor the deterioration process. These intermediate metabolites tended to be transformed into lower-level products involving pyruvate, acetyl coenzyme A, and fumaric acid, used in the tricarboxylic acid cycle, performing substance transformation. This comprehensive analysis of metabolites provides a valuable reference for monitoring the freshness of goose meat, potentially improving the safety of domestic poultry products.

## Simple Summary

Cold chain logistics and chilled poultry product marketing are widely used. Prolonged storage can affect meat quality, so ensuring the quality of chilled poultry meat has attracted worldwide attention. Non-targeted metabolomics can detect small chemical compounds associated with meat quality. This study identified 18 specific metabolites related to the breakdown of carbohydrates, amino acids, nucleotides, and lipids as marker compounds. The results suggest new methods for assessing the freshness of goose meat, thus having the potential to improve food safety in domestic poultry products.

## 1. Introduction

Due to increasing human population sizes, living standards, and the desire for improved health and quality of life, consumers seek a reliable supply of safe and high-quality meat products [1]. The goose, highly regarded for its high protein content, low fat levels, and abundance of unsaturated fatty acids, is becoming increasingly popular among consumers in China, Poland, and other regions that highly produce geese [2]. However, due to outbreaks of H7N9 influenza, live poultry trading was severely restricted in China [3]. Due to the implementation of measures related to cold-chain logistics and effective refrigeration [4], there is a growing preference for buying chilled goose meat through online platforms or supermarkets. Ensuring the quality of refrigerated goose meat has thus become a significant global problem.

Although refrigerated storage is effective for preserving goose meat, the quality of the meat decreases continuously with prolonged storage, which can be seen in reduced odor scores, increases in viable bacteria counts, darkening of the meat color, and even spoilage [5]. This deterioration renders the meat unfit for human consumption, primarily due to visible differences, such as too much slime, color changes, or the appearance of different odors [6]. This spoilage process is attributed to a cascade of biochemical events involving lipid oxidation, autolytic enzymatic reactions, and microbial activity, generating a spectrum of low molecular weight metabolites [7]. The analysis and characterization of these metabolites would thus yield invaluable insights for modulating and evaluating meat quality [8].

Untargeted metabolomics can analyze changes caused by small chemicals (<1500 Da) and interpret changes in the quality characteristics of meat under different environmental conditions, and its use has thus become increasingly popular in meat science. This is achieved using sophisticated analytical techniques [9]. Ultra-high-performance liquid chromatography-tandem mass spectrometry (UHPLC-MS/MS) is a detection technique known for its speed of separation, high sensitivity, and selectivity. It plays a significant role in identifying the quality and safety of meat [10]. In the context of research on pork [11], beef [12], duck [13], and chicken [14] meat, metabolomics has proven invaluable in uncovering the dynamic shifts in metabolic constituents and elucidating the mechanisms governing the degradation of livestock meat during storage. However, the relationship between changes in non-volatile metabolites and metabolic pathways in geese remains poorly understood.

To bridge this knowledge deficit, the present study utilized untargeted metabolomics with UHPLC-MS/MS to identify potential marker compounds and their effects on the metabolism of non-volatile substances during the storage of goose meat. The results offer a theoretical foundation for identifying different metabolites linked to meat quality during prolonged storage. Additionally, they contribute to developing novel biomarkers for assessing the freshness of refrigerated goose meat.

## 2. Materials and Methods

### 2.1. Ethics Approval

The investigation underwent thorough scrutiny and received approval from the Institutional Animal Care and Use Committee within the Department of Animal Science and Technology at Yangzhou University (Approval number SYXK [Su] 2016-0020, China). The handling of geese followed the 2008 Standards for the Administration of Experimental Practices (Jiangsu, China) in a precise manner.

### 2.2. Sample Preparation

Yangzhou geese were raised under uniform conditions at a commercial goose farm in Taizhou, Jiangsu, China. Thirty-two geese, aged 70 days, were randomly selected from a larger flock comprising 1000 birds. These geese were of the same age and had the same feeding program. The birds were euthanized using humane methods, specifically by severing the jugular vein and carotid artery on one side of the neck. Afterward, the muscle tissue from the middle position of the right breast along the sternum was surgically removed using a scalpel. After the trimming of visible fat and connective tissue, the meat samples were placed separately in clean polyethylene bags, placed on ice, and transported to the laboratory within 30 min. Each breast muscle of each animal was randomly divided into four equal sections (approximately 5 × 4 × 3 cm, 50 g in weight). The meat samples were then stored in a 4 °C refrigerator for 0, 3, 6, and 9 days (D0, D3, D6, and D9). Each group comprised eight replicates. When the storage period was reached, all samples were promptly stored in batches within a freezer set at –80 °C for preservation before further analysis.

### 2.3. Metabolite Extraction

Three points were selected on the muscle samples, and approximately 25 mg of breast tissue was removed and extracted by the addition of 800 µL of a pre-cooled extraction solution of methanol, acetonitrile, and water in a 2:2:1 (*v*/*v*/*v*) ratio. In addition, the internal standards mix 1 and 2 were combined to improve the accuracy of quality control (QC). The meat was homogenized for 5 min with a TissueLyser (JXFSTPRP, Shanghai, China), the samples were sonicated for 10 min and then kept at −20 °C for 1 h. After centrifugation (25,000 rpm, 15 min, 4 °C), the supernatants were lyophilized under vacuum. The material was then reconstituted in 200 µL of 10% methanol and sonicated for a further 10 min at 4 °C. After centrifugation (25,000 rpm, 15 min), the supernatants were transferred to autosampler vials for LC-MS analysis. In addition, a QC sample was produced by combining equal amounts of each sample.

### 2.4. Ultra-High-Performance Liquid Chromatography-Tandem Mass Spectrometry Analysis

A high-resolution mass spectrometer Q Exactive (Thermo Fisher, Waltham, MA, USA) was used to perform untargeted metabolomics analysis. UHPLC-MS/MS data were collected in both positive and negative ion modes to expand the range of detectable metabolites. Compound Discoverer 3.1 software (Thermo Fisher) was used for subsequent data processing, including peak extraction, alignment, and compound identification. The metaX package in R (2023.12.0+369) and a specific metabolome bioinformatics analytical pipeline were used for comprehensive analysis, including data preparation, statistical assessments, metabolite classification annotations, and functional annotations.

### 2.5. Bioinformatics and Statistical Analyses

After completing the data preparation, the raw data underwent dimensionality reduction using principal component analysis (PCA) to examine the dataset’s groupings, trends, and outliers. Subsequently, partial least squares-discriminant analysis (PLS-DA) was utilized, using the variable importance in projection (VIP) values of the first two principal components, along with variability analysis, fold-change criteria (fold-change > 1.2 or fold-change < 0.83), and *t*-tests (*p* < 0.05) to identify differential metabolites. These analytical steps were performed using Simca 14.1 software. MetaboAnalyst 6.0 software (https://www.metaboanalyst.ca/, accessed on 28 December 2023) was used for visual representations of the differences in metabolite contents across goose samples preserved for different lengths of time through heatmap analysis. In addition, violin plots were drawn for a visual representation of significant variations in metabolite dynamics. Random forest curves were generated using MATLAB R2023b. Furthermore, the metabolic pathways associated with the identified compounds were identified using the Kyoto Encyclopedia of Genes and Genomes (KEGG) pathway database (*p* < 0.05). Before analysis, all samples underwent log transformation and auto-scaling.

## 3. Results and Discussion

### 3.1. Quality Control and Principal Component Analysis of Components in Goose Breast Meat Samples

The study employed the UHPLC-MS/MS to analyze changes in metabolite profiles in goose breast muscle samples during storage. Supplementary Figure S1 illustrates the congruent base peak chromatograms in the positive and negative ion modes, showing satisfactory spectral overlap and consistent peak response intensity. These results highlight the instrument’s excellent performance and consistent signal stability during sample detection and processing. A total of 2919 compounds were initially detected in goose samples at different storage durations using the positive and negative ion modes. Following stringent filtration criteria, a subset of 1226 compounds, comprising 819 metabolites in the positive ion mode and 407 in the negative ion mode, were successfully identified through qualitative analysis.

**Figure 1:**
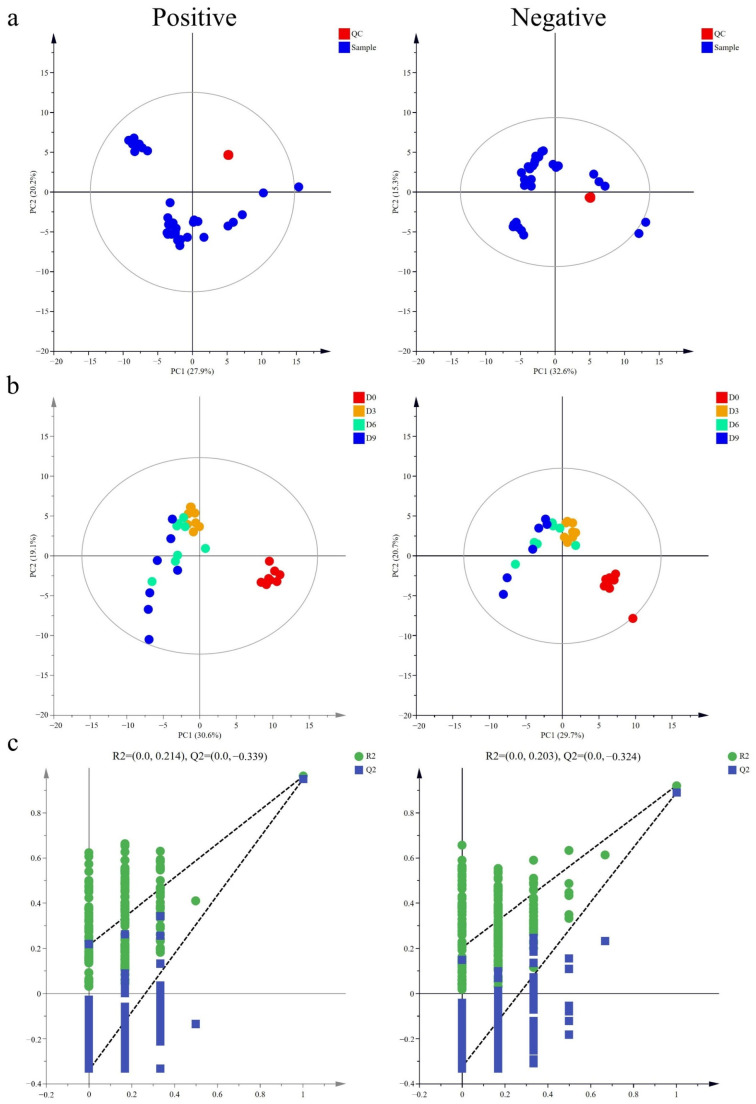
Quality control (QC) and partial least squares discriminant analysis (PLS-DA) of metabolomic data from goose breast meat samples. (**a**) Principal component analysis (PCA) of QC samples in the positive and negative ion modes. The abscissa represents the first principal component, and the ordinate represents the second principal component. (**b**) PLS-DA models based on the normalized metabolomics data under the positive and negative ion modes. Each point in the graph represents one sample, and samples from the same group are shown in the same color. The distance between each point indicates the difference between the metabolites in the sample. (**c**) Permutation plot used to verify the PLS-DA model under positive and negative ion modes. The two dashed lines represent the regression lines of R2 and Q2 respectively. Abbreviations: D0, refrigerated storage for 0 days; D3, refrigerated storage for 3 days; D6, refrigerated storage for 6 days; D9, refrigerated storage for 9 days.

PCA, an unsupervised technique for reducing dimensionality, was used to identify complex patterns in the metabolite data from refrigerated meat samples stored for different durations [15]. As illustrated in Figure 1a, this showed a robust PCA model for comparative analysis, revealing a cohesive clustering of all QC samples. This tight clustering underscores the robust stability of the UHPLC-MS/MS system throughout the analytical process.

In contrast to the unsupervised PCA method, PLS-DA operates as a supervised statistical approach, providing increased sensitivity for exploring differences among classification groups. The PLS-DA outcomes showed marked differences between fresh meat samples (D0) and those subjected to prolonged storage durations (D3, D6, and D9) (Figure 1b). With the extension of the storage length, a clear separation from the fresh sample group (Group D0) became evident, highlighting the significant influence of longer storage periods on metabolic profiles [16]. Moreover, a distinct pattern was observed where longer storage times led to increased distances between samples in the same group [17], which could indicate progressive decomposition during storage. In an attempt to determine the model’s resilience against overfitting, a permutation plot was established. As shown in Figure 1c, all Q2 points consistently fell below the rightmost original Q2 point, with the regression line of Q2 dipping below zero at the ordinate intersection, confirming the superior predictive capacity of the established model.

### 3.2. Identification of Differential Metabolites

**Figure 2:**
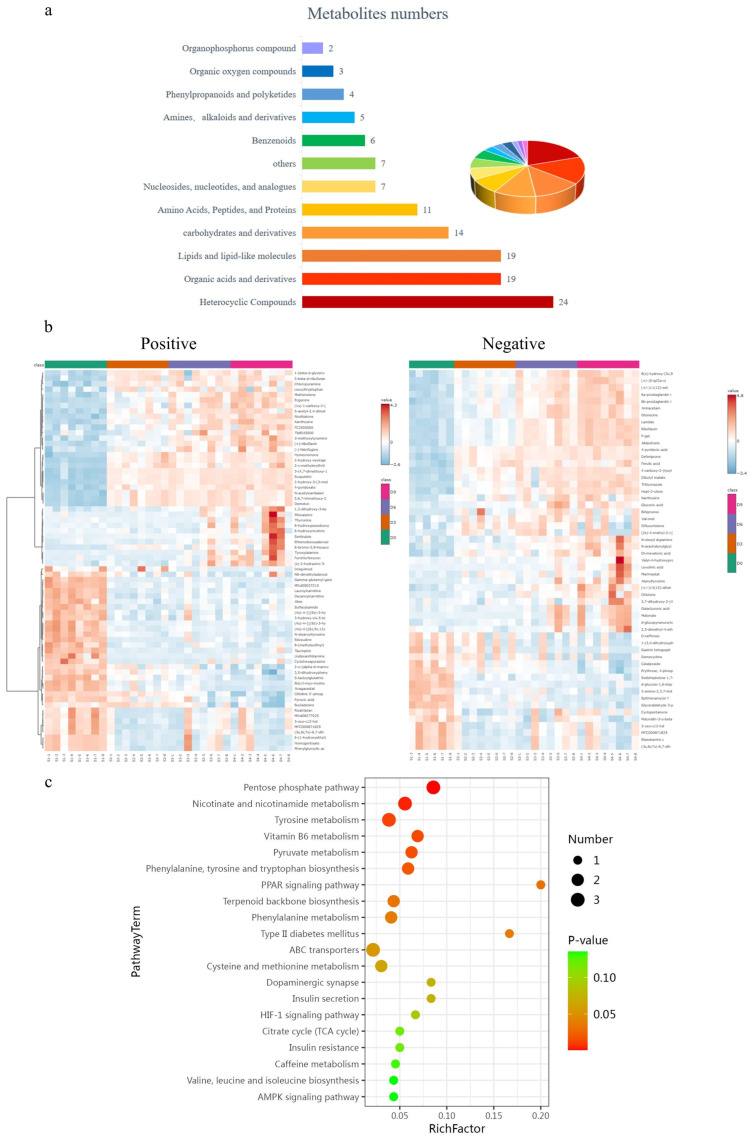
Abundance of differential metabolites in goose meat at different time points during storage. (**a**) Types of differential metabolites in goose meat. (**b**) Heatmap of hierarchical clustering analysis of metabolite variations identified in the positive and negative ion modes. (**c**) The top 20 pathways identified by the Kyoto Encyclopedia of Genes and Genomes (KEGG) enrichment of the differential metabolites at different time points during storage.

The PLS-DA model was used to determine the variations in metabolite abundance within the geese samples. Significant differences between groups were determined using the criteria described above. The differential metabolites in positive and negative ion modes were designated for 121 of the 1226 metabolites. These metabolites belonged to various classes, including heterocyclic compounds (19.83%), organic acids and derivatives (15.7%), lipids and lipid-like molecules (15.7%), carbohydrates and derivatives (11.57%), amino acids, peptides, and proteins (9.10%), nucleosides, nucleotides, and analogs (5.79%), others (5.79%), benzenoids (4.96%), amines, alkaloids, and derivatives (4.13%), phenylpropanoids and polyketides (3.31%), organic oxygen compounds (2.48%), and organophosphorus compounds (1.65%) (Figure 2a).

A heatmap was utilized to depict the 121 differential metabolites (Figure 2b), revealing that the D0 and D9 time points exhibited the most pronounced increases and decreases in metabolite levels, thereby indicating alterations in various metabolite concentrations over prolonged storage [18].

To elucidate the primary changes in metabolic pathways during the extended storage of goose meat, KEGG enrichment analysis was conducted on the differential metabolites identified between Groups D0 and D9. Figure 2c illustrates the top 20 most significantly enriched metabolic pathways, encompassing those related to carbohydrates, amino acids, vitamins, lipids, and nucleotides. These pathways included essential functions such as the ABC transporters, tyrosine metabolism, vitamin B6 metabolism, the peroxisome proliferator-activated receptors (PPAR) signaling pathway, and the pentose phosphate pathway, among others. These pathways have been documented to be involved in the degradation of nutrients and the generation of metabolic by-products [19,20,21]. Therefore, it may be deduced that carbohydrates, lipids, nucleotides, and other vital elements present during cold preservation of meat undergo enzymatic degradation when exposed to oxygen, leading to a reduction in nutrients [22].

### 3.3. Identification of Potential Marker Compounds in Refrigerated Goose

**Figure 3:**
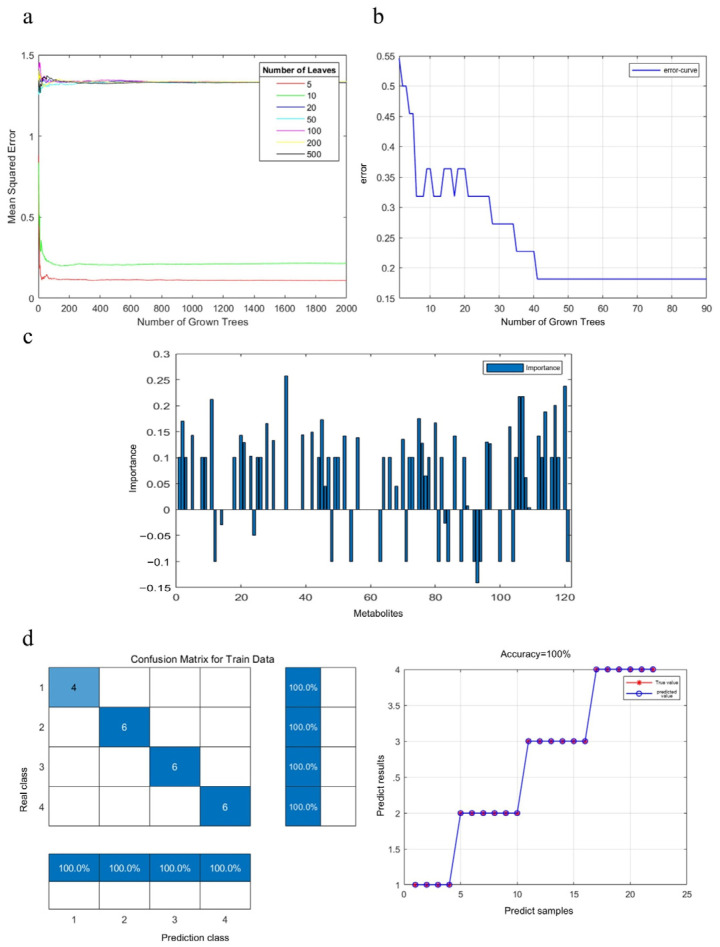
Potential metabolite biomarkers in goose breast meat, identified by random forest regression. (**a**) Cumulative error rates in the random forest classification. (**b**) The mean square error analysis was based on the number of trees and leaves. (**c**) Listing of metabolic biomarkers in order of importance. (**d**) Confusion matrix and prediction accuracy of train set data.

As a machine learning algorithm, the random forest model employs a regression tree technique that achieves high prediction accuracy through the use of bootstrap aggregation and predictor randomization [23]. The random forest model has been extensively used to predict metabolite changes [24,25], offering unique advantages in this field. A rigorous random forest model was used to identify significant variations in compounds in the goose meat samples [26]. The configuration entailed setting the classification trees for the 121 metabolites to 90, with a stipulated minimum of five leaves per tree (Figure 3a). Throughout the construction of these trees, one-third of the samples were deliberately excluded from the bootstrap sample, constituting the out-of-bag data. The out-of-bag data were later used as test samples to obtain an unbiased estimate of the classification error, referred to as the out-of-bag error. After the generation of many trees, a significant decrease in the cumulative out-of-bag error rates was observed, which were reduced to 0.18 (Figure 3b). Furthermore, the model exhibited high reliability, as evidenced by the confusion matrix (Figure 3d). Based on the importance of the out-of-bag data predictor, 27 key metabolites were identified (Figure 3c; Supplementary Table S1). Of these, carbohydrates and derivatives, as well as heterocyclic compounds, formed the greatest proportion, followed by amino acids, peptides, proteins, lipids, lipid-like compounds, organic acids, and derivatives were observed.

To determine the major metabolic biomarkers, the differential metabolites were clustered using a time series trend analysis to assess their expression patterns (Supplementary Figure S2). Attention was given to metabolites that showed patterns of continuous increase and decrease within clusters, as these systematic changes were more likely to be affected by the prolongation of storage time [27]. This showed that the relative abundance of 41 metabolites increased over the refrigerated storage period while the relative abundance of 31 metabolites decreased. These reductions could plausibly be linked to the proliferation of spoilage microorganisms, metabolizing the nutritional substrates in goose meat to generate metabolic by-products [28]. On the other hand, metabolites that increased in abundance could represent the metabolic consequences of degradation, thereby adding to the deterioration of the meat.

### 3.4. Dynamic Changes in Key Metabolites During Long-Term Storage of Goose Breast Meat

In order to better understand the differences in metabolites between fresh and chilled goose, an integrated analysis was conducted to determine if these crucial metabolites could be used as reliable indicators to assess the freshness of chilled goose meat. This analysis included compounds that displayed properties that met the specific random forest criteria. These compounds also showed increases or decreases in relative abundance with the prolongation of storage duration. The abundance of the 18 metabolite biomarkers was analyzed to understand changes in metabolism occurring at different time points (Supplementary Table S2). The results revealed that 14 specific metabolites increased in abundance, while the abundance of 4 decreased during storage. The metabolites that were reduced might serve as substrates utilized by spoilage bacteria to generate metabolic by-products, thereby enriching metabolites in the refrigerated meat, potentially indicating deterioration of the meat.

**Figure 4:**
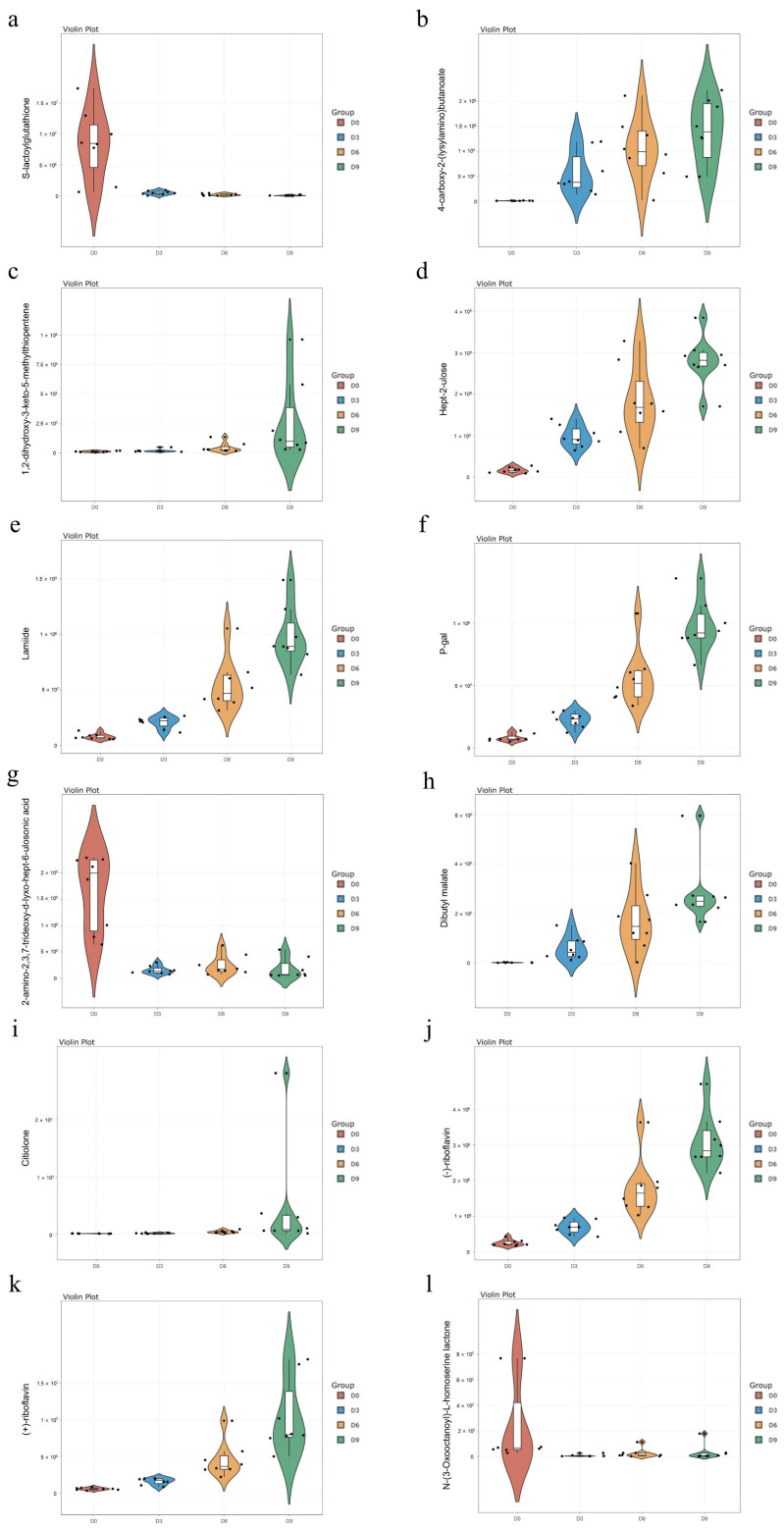
Violin plots of 12 metabolites identified in goose breast meat over storage using the random forest model and time series analysis. The group division is indicated on the *x*-axis of violin plots. The *y*-axis of violin plots represents the relative abundance of metabolites. The metabolites depicted in (**a**–**l**) are as follows: S-lactoylglutathione; 4-carboxy-2-(L-lysylamino)butanoate; 1,2-dihydroxy-3-keto-5-methylthiopentene; Hept-2-ulose; Lamiide; P-gal; 2-amino- 2,3,7-trideoxy-d-lyxo-hept-6-ulosonic acid; Dibutyl malate; Citiolone; (-)-riboflavin; (+)-riboflavin; N-(3-Oxooctanoyl)-L-homoserine lactone.

During refrigeration, the breakdown of proteins in the meat results in the formation of small peptides, which are subsequently degraded to amino acids [29]. These peptides are pivotal in determining the quality of the meat during cold storage. S-lactylglutathione (an oligopeptide) was observed to decrease during storage (Figure 4a). At the same time, dipeptides with lower molecular weights, such as 4-carboxy-2-(lysylamino) butanoate, tended to increase in abundance with prolongation of the storage time (Figure 4b), suggesting accelerated protein degradation during storage. Furthermore, 1,2-dihydroxy-3-keto-5-methylthiopentene (Figure 4c), a member of the dioxygenase family, increased in abundance with prolonged storage time, indicative of enhanced biological oxidation catalyzed by this compound [30].

The current study focused on the dynamic changes in carbohydrates, stored explicitly as “glycogen” in muscle tissues, as emphasized by Ahmad et al. [31]. Notably, the abundance of three specific sugars, namely, hept-2-ulose, lamiide, and p-gal, increased as the duration of storage extended (Figure 4d–f). Furthermore, reduced levels of 2-amino-2,3,7-trideoxy-d-lyxo-hept-6-ulosonic acid, a ketone derivative (Figure 4g) involved in metabolic processes and the maintenance of redox states, were observed, suggesting a retardation in metabolic activity in the meat over time [32]. The microbial breakdown of carbohydrates in meat represents a source of energy, producing water, carbon dioxide, organic acids, and other by-products. The levels of two organic acids, namely dibutyl malate and citiolone (Figure 4h,i), gradually increased with the extension of storage time.

Vitamins are integral to many metabolic pathways, encompassing a cascade of chemical and biochemical reactions. Riboflavin, as demonstrated by Suwannasom et al. [33] and Olfat et al. [34], exhibits diverse functions in metabolism. Theoretical degradation of riboflavin derivatives during prolonged cold storage [35]. In the current study, two different types of riboflavin were observed in chilled goose samples. These forms consistently increased in concentration over the storage period, as shown in Figure 4j,k. Hunt [36] documented a noticeable increase in riboflavin levels with meat preservation. The differences between these results may be due to differences in the methods used to prepare the samples, ultimately affecting the measured levels of the metabolites [37].

Fat degradation entails three primary processes, namely, oxidation, hydrolysis, and anaerobic deterioration [27]. Fat eventually breaks down through these pathways into aldehydes, ketones, low-grade fatty acids, sulfide, indole, methyl ketones, and low molecular weight fatty acids [38]. Among these compounds, four lipids and lipid-like molecules showed increasing abundance with the extended storage duration, indicating accelerated fat degradation rates over time. N-(3-Oxooctanoyl)-L-homoserine lactone is an intermediate signaling molecule in bacterial quorum sensing [39]. After reaching the threshold, its concentration gradually declined in storage, suggesting an escalation in bacterial aggregation (Figure 4l).

### 3.5. The Potential Metabolic Process of Goose Meat During Long-Term Storage

**Figure 5:**
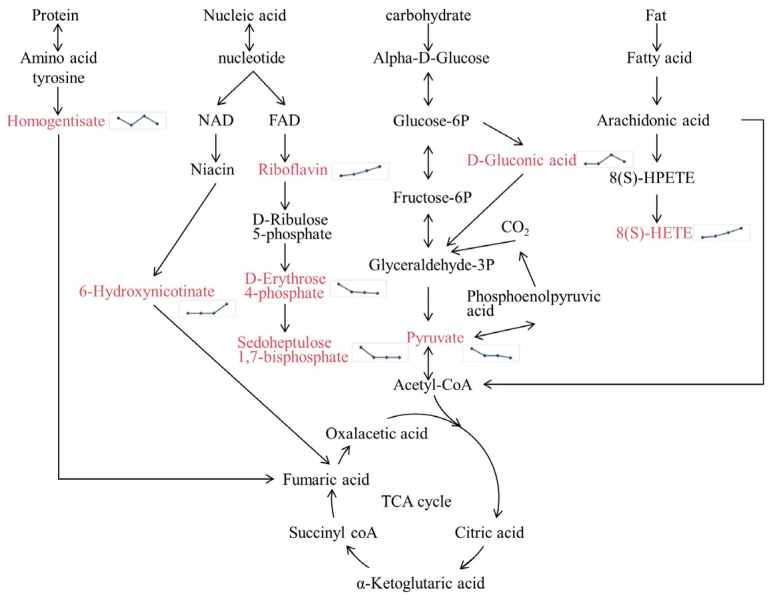
The metabolic pathways involved in goose meat during long-term storage. The line chart indicates the relative contents of metabolites at different storage times. The dots represent the time points D0, D3, D6, and D9, from left to right. The highlighted areas represent differential metabolites. Abbreviations: NAD, nicotinamide adenine dinucleotide; FAD, flavin adenosine dinucleotide; Acetyl-CoA, acetyl coenzyme A.

In order to fully understand the processes controlling the cold storage of goose breast over time, the complex biochemical pathways of the metabolites were investigated (Figure 5). Goose breast meat contains various compounds involved in the breakdown of carbohydrates during storage. These compounds can induce changes in small carbohydrate molecules and their derivatives through glucose degradation involving glycolysis, pyruvate oxidation, and the pentose phosphate pathway [40]. Pyruvate, a pivotal metabolite in glycolysis [41], was observed to decrease in abundance, potentially impeding its conversion to acetyl coenzyme A (acetyl CoA), which could be subsequently engaged in the tricarboxylic acid (TCA) cycle [42]. Proteins undergo hydrolysis, releasing peptides and amino acids [43], further degrading within goose meat during storage. Metabolic fluctuations associated with amino acid degradation indicated changes in the homogentisate content, potentially influencing metabolic dynamics through the involvement of fumaric acid in the TCA cycle [44]. Both 6-hydroxy nicotinate and riboflavin production increased as the storage period increased from D0 to D9. However, the levels of D-erythrose 4-phosphate and sedoheptulose 1,7-bisphosphate decreased. Dramatic changes in nucleic acids and their derivatives were observed over storage for long periods. Fat degradation in meat may be accelerated by spoilage, leading to increased formation of glycerides and fatty acids [45]. The content of 8(S)-HETE increased progressively during storage, activating the PPAR pathway and modulating lipid metabolism [46]. This metabolite decomposes further to form acetyl CoA that participates in the TCA cycle. In addition, metabolic pathways associated with bacterial activity, such as carbon fixation pathways including the Calvin cycle, 3-hydroxypropionate bi-cycle, and dicarboxylate-hydroxybutyrate cycle, showed increased levels of activity.

As previously described, intermediate metabolites, such as those circulating through the TCA cycle, may influence substances within metabolic processes. These intermediates generally tend to convert products to lower levels. Although goose breast meat is maintained at a moderate temperature, the nutrients are susceptible to bacterial consumption and deterioration with the extension of the preservation period.

## 4. Conclusions

This study investigated changes in the relative abundance of metabolites in goose meat during extended storage conditions using non-targeted metabolomics. The outcomes revealed a comprehensive metabolomic profile comprising 121 differential metabolites, signifying significant alterations in the meat composition on prolonged storage. After careful analysis, 18 specific metabolites related to the breakdown of carbohydrates, amino acids, nucleotides, and lipids were identified as marker compounds. These intermediate metabolites can transform into one another through the TCA cycle. The results present a comprehensive analysis of metabolites and suggest new methods for monitoring the freshness of goose meat. Nutrient loss was apparent within 9 days of refrigeration, suggesting that the optimal refrigeration time for goose meat should not exceed 9 days.

## Data Availability

All the data supporting the results are included in the article. The dataset is available from the corresponding author upon reasonable request.

## Supplementary Materials

The following supporting information can be downloaded at: https://www.mdpi.com/article/10.3390/vetsci11120637/s1, Figure S1. A visual representation of the congruent base peak chromatograms in positive and negative ion modes. Figure S2. Cluster diagram of time series trend analysis of compounds. Table S1. 27 key metabolites based on the importance of the OOB predictor. Table S2. 18 metabolite biomarkers.
