# Supplementary figures and images for: Non-Targeted Metabolomics Analysis of Small Molecular Metabolites in Refrigerated Goose Breast Meat
Source: Vet Sci. 2024 Dec 9;11(12):637. doi: 10.3390/vetsci11120637 (PMC11680168; doi:10.3390/vetsci11120637)

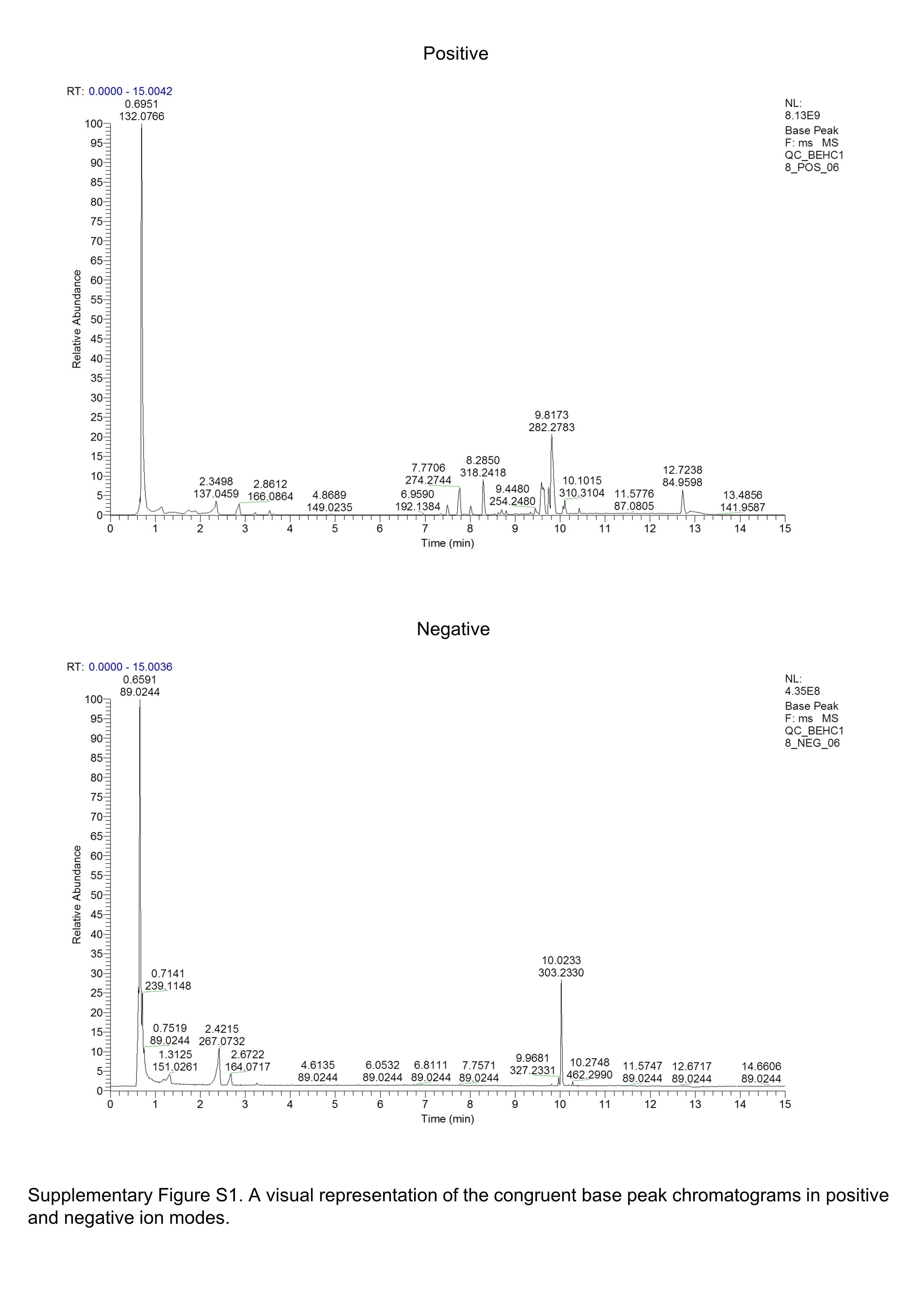

Supplement: Supplementary file 1 [file vetsci-11-00637-s001.zip › Supplementary Figure S1.jpg]

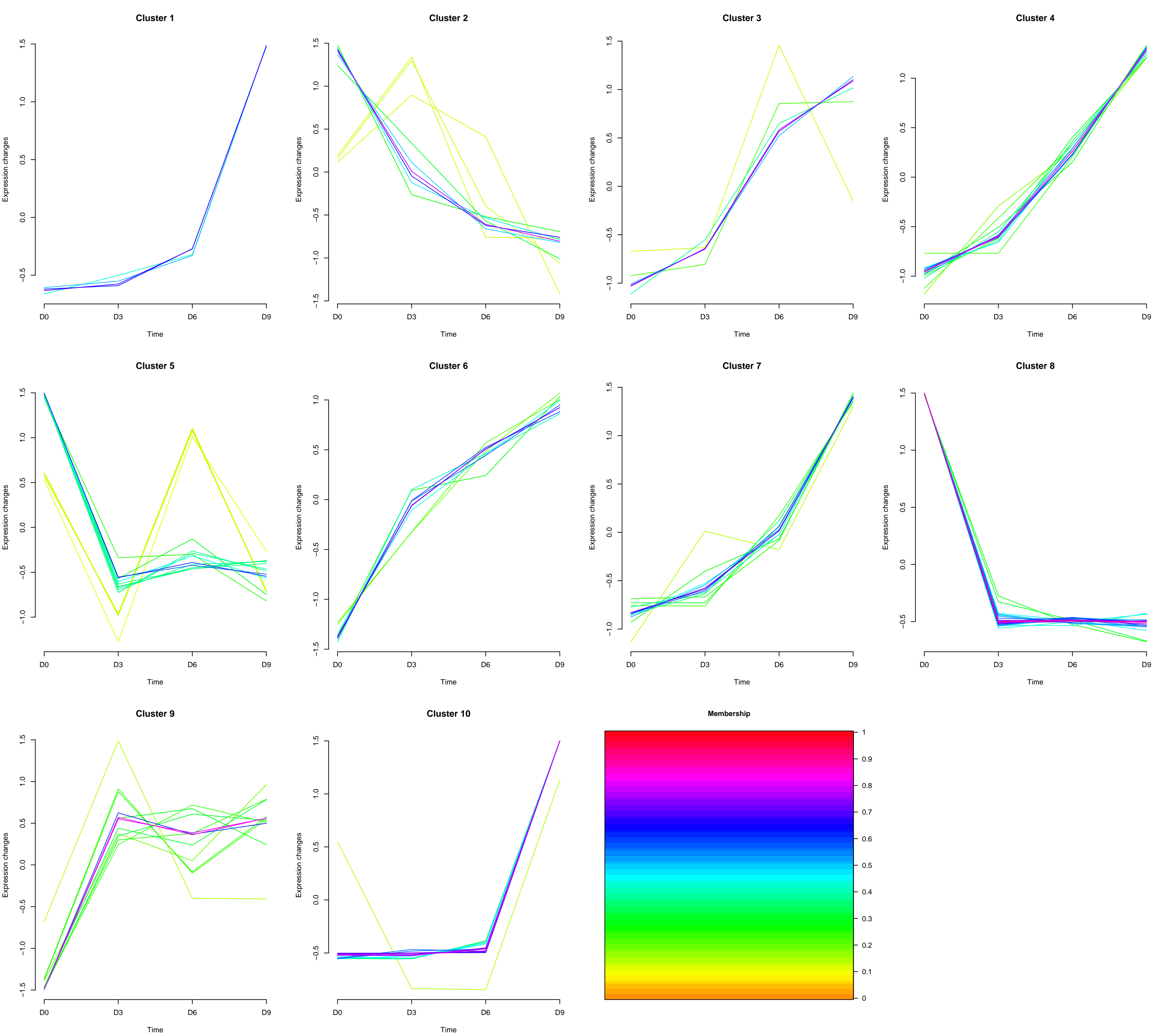

Supplement: Supplementary file 1 [file vetsci-11-00637-s001.zip › Supplementary Figure S2.pdf]
